# Systematic Characterization of Circular RNA-Associated CeRNA Network Identified Novel circRNA Biomarkers in Alzheimer's Disease

**DOI:** 10.3389/fbioe.2019.00222

**Published:** 2019-09-11

**Authors:** Yan Zhang, Fulong Yu, Siqi Bao, Jie Sun

**Affiliations:** School of Ophthalmology & Optometry and Eye Hospital, School of Biomedical Engineering, Wenzhou Medical University, Wenzhou, China

**Keywords:** Alzheimer's disease, circular RNA, competing endogenous RNA, ceRNA network, system biology

## Abstract

Alzheimer's disease (AD), a degenerative disease of the central nervous system, is the most common form of dementia in old age. The complexity and behavior of circular RNA (circRNA)-associated competing endogenous RNA (ceRNA) network remained poorly characterized in AD. The aim of this study was to elucidate the regulatory networks of dysregulated circRNAs from ceRNA view and identify potential risk circRNAs involved in AD pathogenesis. Consistent differentially expressed genes (CDEGs) were obtained using meta-analysis for multiple microarrays, and differentially expressed miRNAs (DEmiRs) were identified using empirical Bayes method. The circRNA-associated ceRNA network (cirCeNET) was constructed based on “ceRNA hypothesis” using an integrated system biology method. A total of 1,872 CDEGs and 48 DEmiRs were screened across different datasets. By mapping CDEGs and DEmiRs into the cirCeNET, an AD-related circRNA-associated ceRNA network (ADcirCeNET) was constructed, including 3,907 edges and 1,407 nodes (276 circRNAs, 14 miRNAs and 1,117 mRNAs). By prioritizing AD risk circRNA-associated ceRNAs, we found that the circRNA *KIAA1586* occurred most frequently in the AD risk circRNA-associated ceRNAs and function as a ceRNA that operates by competitively binding three known AD-risk miRNAs. *In silico* functional analysis suggested that circRNA *KIAA1586*-related ceRNA network was significantly enriched in known AD-associated biological processes. Our study provided a global view and systematic dissection of circRNA-associated ceRNA network. The identified circRNA *KIAA1586* may be a key risk factor involved in AD pathogenesis.

## Introduction

Alzheimer's disease (AD), a degenerative disease of the central nervous system, is the most common form of dementia in old age (Jiang et al., 2013; Liu et al., 2019). It is mainly manifested as neuropsychiatric symptoms such as progressive memory disorder, cognitive dysfunction, personality change, and language disorder, which seriously affect social, professional and life functions. With the increasing morbidity year by year, AD has attracted more attention from society. However, the etiology and pathogenesis of AD have not been well-elucidated yet.

Currently, the rapid advancement of high-throughput technologies offers great opportunities for biomarker identification (Liu et al., 2018a,b; Yu et al., 2018a). Non-coding RNAs as biomarker and therapeutic targets play a significant role in human diseases (Long et al., 2012; Zhou et al., 2015a,b, 2016, 2017, 2018a,b,c; Yu et al., 2018b). Circular RNAs (circRNAs) are a naturally occurring class of non-coding RNA molecules, which has become the latest research focus in the field of RNA (Qu et al., 2015). Unlike traditional linear RNA, circRNA has a closed circular structure and is not affected by RNA exonuclease, so its expression level is more stable and not easily degraded (Li et al., 2015). However, classical RNA sequencing methods can only isolate those molecules with polyA tails. The ends of circRNAs are attached together, lacking these “tails,” and are generally ignored. In fact, hundreds of circRNAs are enriched in the mammalian brain tissues (Hansen et al., 2013; Rybak-Wolf et al., 2015) and have important regulatory potency (Memczak et al., 2013). But a key question remains: what do they actually do?

The occurrence of complex diseases is the result of synergism of multiple interacting genes or RNAs. Therefore, we should investigate disease mechanism at the level of system biology and mine useful information from a vast network of interacting genes or RNAs (Zhang et al., 2018). Integration analysis of transcription (protein-coding genes) and post-transcriptional (non-coding genes) regulation has proven to be a valuable strategy for studies of the genetic characteristics of various human complex diseases (Zhang et al., 2015; Arena et al., 2017). In a recent study, Welden et al. found the human MAPT gene which produces the microtubule-associated protein Tau, generates circRNAs to contribute to AD (Welden et al., 2018). Piwecka et al. (2017) demonstrated that loss of circRNA locus affected brain function. They showed circRNA *Cdr1as* functions as miRNA sponge by binding miRNA response elements (MREs) to cause miRNA deregulation. In addition, Lukiw (2013) revealed that circRNA *ciRS-7* also acts as a ceRNA to absorb miRNA. A deficiency in *ciRS-7* “sponging” effects might be expected to increase the expression level of miR-7 in AD-affected brain cells and down-regulate AD-relevant targets. These studies suggest that such circRNA-miRNA-mRNA competing system is an important epigenetic regulatory layer control over gene expression in AD (Salmena et al., 2011; Zhang et al., 2018). However, the complexity and behavior of circRNA-associated competing endogenous RNA (ceRNA) network remain poorly characterized in the pathogenesis of AD.

Therefore, in this study, by comprehensively integrating gene and miRNA expression data of AD, the AD-related circRNA-miRNA-mRNA competitive network (ADcirCeNET) was established. And then, AD risk circRNA-miRNA-mRNA relationships were optimized using the known AD-related data resources. We found circRNA *KIAA1586* could contribute to AD. Our results showed that *KIAA1586* acts as a ceRNA to absorb three miRNAs (*hsa-miR-29b, hsa-miR-101, hsa-miR-15a*) and lead to the dysregulation of AD-associated biological processes.

## Materials and Methods

### Differential Expression Analysis of Gene and miRNA in AD

We obtained four gene expression profiles of AD from Gene Expression Omnibus (GEO) database, their accession ID were GSE5281 (Liang et al., 2007), GSE1297 (Blalock et al., 2004), GSE12685 (Williams et al., 2009), and GSE16759 (Nunez-Iglesias et al., 2010), respectively. Since the brain is the most complex part of all human organs, it can be divided into multiple regions with different functions. In this study, the gene expression data detected six different brain regions including entorhinal cortex, hippocampus, superior frontal gyrus, posterior cingulated, medial temporal gyrus, and primary visual cortex. To discover the common pathogenic mechanism in different brain regions, the gene expression profiles were divided into six parts according to the brain regions for separate analysis. All of the data were normalized and log2 transformed. The microarray probe IDs were converted to Entrez Gene IDs. To obtain the CDEGs, the differentially expressed gene lists were combined using the R package “metaMA” (Marot et al., 2009). The miRNA expression profile (accession ID GSE16759) was derived from the GEO database, including four AD samples and four normal controls. The DEmiRs were identified using empirical Bayes method (Nunez-Iglesias et al., 2010).

### Construction of circRNA-miRNA-mRNA Competitive Network

It has been reported that circRNA dysfunction can lead to the occurrence of AD (Lukiw, 2013), and the main way of its function is competitive regulation (Qu et al., 2015). Here, a competitive network among circRNA, miRNA and mRNA was constructed. First, we downloaded the human miRNA-circRNA and miRNA-mRNA interactions from the RAID database which is a resource of RNA-associated interactions across organisms (Yi et al., 2017). A necessary condition for competitive regulation is the number of shared miRNAs (Zhang et al., 2018). And then, the miRNA-circRNA and miRNA-mRNA interactions shared at least five miRNAs were retained. Finally, all these identified miRNA-mediated circRNA-mRNA ceRNA crosstalk were integrated to build a circRNA-miRNA-mRNA competitive network (cirCeNET).

### Identification of circRNA-Associated ceRNA Network of AD

The CDEGs and DEmiRs were mapped into the cirCeNET. The circRNA-miRNA-mRNA competitive relationships were extracted which contained at least one CDEG or DEmiR. These competitive relationships constitute the circRNA-associated ceRNA network of AD (ADcirCeNET).

The coverage rate of known AD-associated gene and miRNA set was used to prioritize AD risk circRNA-associated ceRNAs. If a miRNA-mediated circRNA-mRNA ceRNA crosstalk contained at least one known AD-associated gene or miRNA, it was considered as the AD risk circRNA-associated ceRNA.

### Function Enrichment Analysis

The significant enriched biological functions of a gene set were explored using the R package “clusterProfiler” (Yu et al., 2012). The adjusted *P*-values were calculated using the multiple test of Benjamini and Hochberg (BH) method.

## Results

### AD-Related Gene and miRNA Set

To reveal the common pathogenesis of AD, the gene expression profiles were split into six parts according to different brain region. Through integrated differential expression analysis across different datasets, 1872 CDEGs were screened under FDR < 0.01. The CDEG list can be seen in Supplementary File 1. For miRNA expression profile, 48 DEmiRs were obtained under FDR < 0.05. The DemiR list can be seen in the Supplementary File 2.

The known AD-associated genes and miRNAs were derived from GeneCards (Safran et al., 2010), HMDD (Lu et al., 2008) and miR2Disease (Jiang et al., 2009) databases which are all manually curated data resources. There were 27 genes and 45 miRNAs.

### The circRNA-Associated ceRNA Network in AD

We derived 7,896 miRNA-circRNA interactions and 719,442 miRNA-mRNA interactions from RAID database. The miRNA-mediated ceRNA crosstalk between circRNA and mRNA were identified by filtering the number of shared miRNA. Finally, 7,120 miRNA-circRNA and 409,381 miRNA-mRNA interactions were obtained and were integrated to build a tremendous cirCeNET. The constructed cirCeNET contained 11,133 nodes (including 484 circRNAs, 280 miRNAs and 10,369 mRNA) and 416,501 edges.

**Figure 1:**
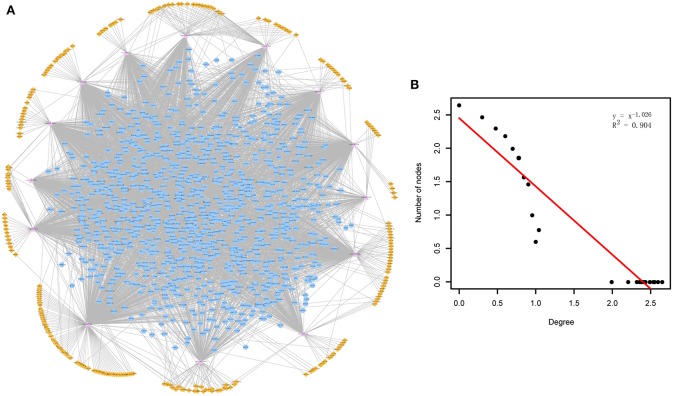
**(A)** The circRNA-associated ceRNA network in AD. The orange diamonds represented circRNAs, purple triangles represented miRNAs and blue circles represented target mRNAs. Network edges represented competitive interactions. **(B)** Degree distribution of the ADcirCeNET.

Through mapping the CDEGs and DEmiRs into the constructed cirCeNET, the miRNA-mediated ceRNA crosstalks were extracted if it contained at least one CDEG or DEmiR. These miRNA-mediated ceRNA crosstalks made up the ADcirCeNET (Figure 1A). There were 3,888 edges including 428 miRNA-circRNA and 3,460 miRNA-mRNA interactions, and 1,407 nodes including 276 circRNAs, 14 miRNAs and 1,117 mRNAs in the ADcirCeNET (details see Supplementary File 3). Moreover, examination of the degree distribution of the ADcirCeNET revealed a powerlaw distribution, showing that the ADcirCeNET was scale-free, similar to most biological networks (Figure 1B).

The coverage rate of known AD-associated gene and miRNA set was used to prioritize AD risk circRNA-associated ceRNAs. If a miRNA-mediated ceRNA crosstalk in the ADcirCeNET contained at least one known AD-associated gene or miRNA, this miRNA-mediated ceRNA crosstalk was considered as the AD risk circRNA-associated ceRNA. Finally, 46,096 AD risk miRNA-mediated ceRNA crosstalk, including 158 circRNAs, 5 miRNAs and 763 mRNAs, were identified.

### Dysregulation of circRNA KIAA1586 Contributes to AD

**Figure 2:**
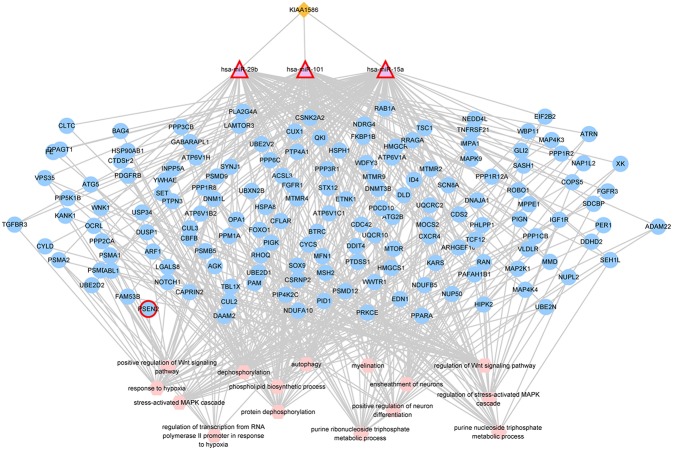
The AD risk circRNA-associated ceRNAs involving in *KIAA1586*. The orange diamonds represented circRNAs, purple triangles represented miRNAs, blue circles represented target mRNAs, and pink hexagons represented GO biological functions. The red border represented known AD-related nodes. The edges among circRNAs, miRNAs and mRNAs represented competitive interactions, and the edges between mRNAs and functions represented that the mRNAs were annotated in the GO terms.

In the AD risk circRNA-associated ceRNAs, the circRNA *KIAA1586* occurred most frequently. The AD risk circRNA-associated ceRNAs involving in circRNA *KIAA1586* were shown in Figure 2 and details can be seen in Supplementary File 4. All of 159 genes and miRNAs were differentially expressed. There are 4 known AD-related genes and miRNAs (PSEN2, hsa-miR-29b, hsa-miR-15a, hsa-miR-101). The crosstalk among *KIAA1586* and AD-risk genes were mediated by hsa-miR-29b, hsa-miR-15a and hsa-miR-101. Thus, we speculate that hsa-miR-29b, hsa-miR-101 and hsa-miR-15a involved in competitive regulation in AD.

The amyloid precursor protein (APP) and its proteolytic product amyloid beta (Aβ) were closely associated with AD (Long and Lahiri, 2011; Long et al., 2019). Aberrant expression and function of miRNAs have been observed in AD and they have important roles in neuropathological conditions. Our results showed that *KIAA1586* could competitive binding three miRNAs (*hsa-miR-29b, hsa-miR-101*, and *hsa-miR-15a*). Pereira et al. (2016) demonstrated that *hsa-miR-29b* is decreased in AD patients displaying over-expression of *hBACE1* and subsequent Aβ peptide. Vilardo et al. (2010) revealed that the inhibition of *hsa-miR-101* increased APP levels and affected the accumulation of Aβ. Hebert et al. (2008) also found that *hsa-miR-15a* was significantly altered in AD brain and predicted that *hsa-miR-15a* regulates APP.

Three miRNAs regulated many genes related to AD. For example, Schlatterer et al. (2011) have shown c-Abl activation in AD and its activation in neuronal culture in response to Aβ fibrils and oxidative stress. Oddo (2012) indicated mTOR signaling lead to the progressive cognitive deficits characteristic of AD. And Peterson et al. (2014) uncovered that variants in *PPP3R1* were associated with rapid functional decline in AD. Thus, our results showed that the dysregulation of circRNA *KIAA1586* might disrupt the balance of three miRNA-related ceRNA networks and contribute to AD.

### Function of KIAA1586-Associated ceRNAs

**Figure 3:**
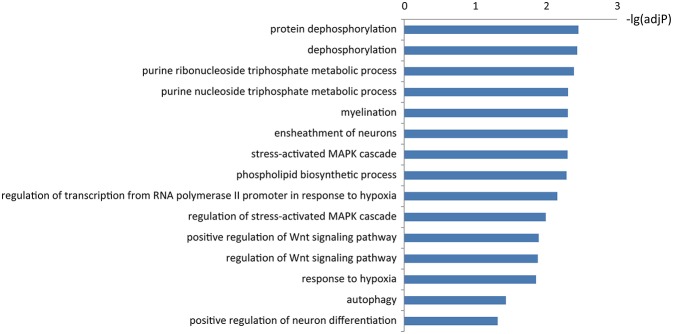
The significantly enriched biological processes of *KIAA1586*-associated ceRNAs.

To learn about the biological functions of KIAA1586-associated ceRNAs, the GO function enrichment analysis (FDR < 0.05) was performed for genes in the circRNA *KIAA1586-*related ceRNA network. We found that mRNAs in the circRNA *KIAA1586-*related ceRNA network were significantly enriched in known AD-associated biological processes (Figure 3, Supplementary File 5), such as Wnt signaling pathway (Boonen et al., 2009), protein dephosphorylation (Goedert et al., 1992), ensheathment of neurons (Morawski et al., 2010), stress-activated MAPK cascade (Morawski et al., 2010) and autophagy (Pickford et al., 2008).

**Figure 4:**
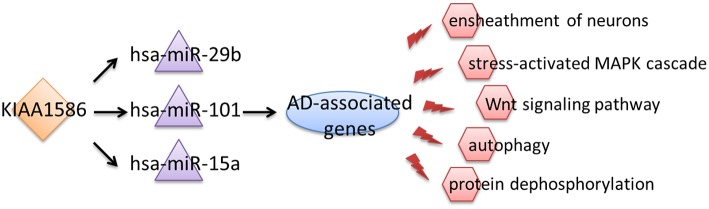
A schematic diagram about circular RNAs, miRNAs, and AD pathologic mechanisms.

Therefore, we inferred that aberrant expression of the circRNA KIAA1586 may be a key risk factor associated with the occurrence and development of AD. Through competitively binding to *hsa-miR-29b, hsa-miR-101* and *hsa-miR-15a*, the expressional perturbation of *KIAA1586* and the resultant changes in *KIAA1586*-associated ceRNA crosstalk interactions cause the abnormalities of AD-associated biological processes and contribute to the risk of AD (Figure 4).

## Discussion

AD is the most common type of dementia and one of the top 10 leading causes of death in the United States (Long et al., 2014; Gupta et al., 2017). The number of people living with AD is projected to nearly triple to 14 million people by 2060. It can seriously affect a person's ability to carry out daily activities. But the cause of AD is poorly understood.

The circRNAs are a class of non-coding RNAs highly expressed in the nervous system. Recent studies showed circRNA to be an important player in the development of neurodegenerative diseases like Alzheimer's disease. Many reports have demonstrated that circRNAs act as a kind of endogenous, competing, anti-complementary miRNA “sponge” to absorb and hence quench normal miRNA functions (Zhang et al., 2018).

In this study, we applied an integrated system biology approach to identify the circRNA-associated ceRNA network in AD. Combining expression information and shared miRNA number, the circRNA-associated ceRNAs were screened through strict threshold setting. We used the target genes of circRNAs to annotate their function through multiple testing (FDR < 0.05). Moreover, through literature review the function annotation was further verified. Our results showed that circRNA *KIAA1586* might contribute to AD and its dysregulation could cause abnormal of AD-related biological functions. Further experimental studies should be conducted to uncover the functional roles of circRNA *KIAA1586* as a potential risk factor in the pathogenesis of Alzheimer. Our method will help to better understand the underlying molecular mechanisms of AD and our results also suggest that circRNA can be taken as a potential biomarker and therapeutic target in AD diagnosis and treatment. With the increasing in available circRNA expression profiles for AD and the accumulation of circRNA regulations or interactions, our method will become more powerful.

## Data Availability

The data analyzed in this study were obtained from the GSE5281 (https://www.ncbi.nlm.nih.gov/geo/query/acc.cgi?acc=GSE5281), GSE1297 (https://www.ncbi.nlm.nih.gov/geo/query/acc.cgi?acc=GSE1297), GSE12685 (https://www.ncbi.nlm.nih.gov/geo/query/acc.cgi?acc=GSE12685) and GSE16759 (https://www.ncbi.nlm.nih.gov/geo/query/acc.cgi?acc=GSE16759).

## Author Contributions

JS conceived and designed the experiments. YZ, FY, SB, and JS performed the experiments and analyzed the data. YZ and JS wrote the paper. All authors read and approved the final manuscript.

### Conflict of Interest Statement

The authors declare that the research was conducted in the absence of any commercial or financial relationships that could be construed as a potential conflict of interest.

## Supplementary Material

Supplementary File 1 The consistent differentially expressed gene list.

Supplementary File 2 The differentially expressed miRNA list.

Supplementary File 3 The AD-related circRNA-associated ceRNA network.

Supplementary File 4 The circRNA KIAA1586-associated ceRNAs.

Supplementary File 5 The function of KIAA1586-associated ceRNAs.
